# Supplementary material for: The Identification of AMT Family Genes and Their Expression, Function, and Regulation in Chenopodium quinoa
Source: Plants (Basel). 2024 Dec 17;13(24):3524. doi: 10.3390/plants13243524 (PMC11676291; doi:10.3390/plants13243524)
Supplement: Supplementary file 1 [file plants-13-03524-s001.zip › Plants-3290654-Supplementary Figures.pdf]

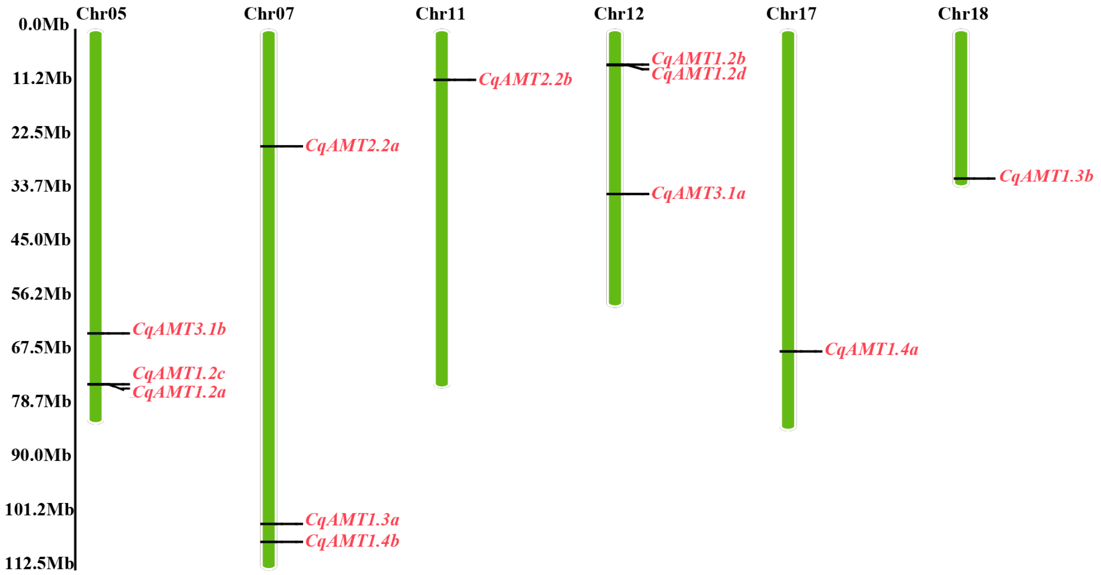

Figure S1. Distribution of *CqAMTs* on the chromosomes and gene duplication of *CqAMTs*.

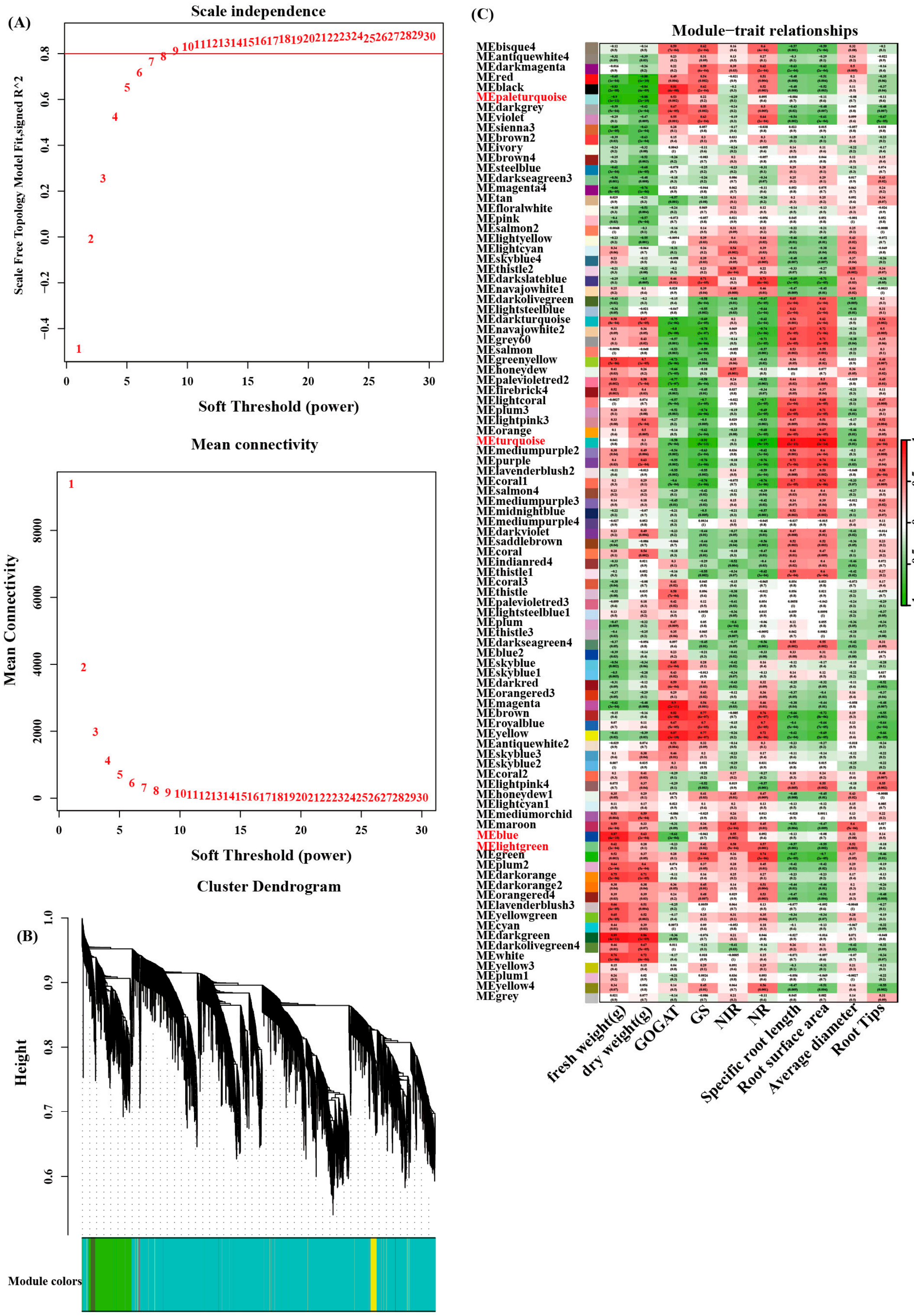

Figure S2. Identification of modules by weighted gene co-expression network analysis (WGCNA). **(A)** Selection of optimal thresholds. **(B)** Constructing a cluster dendrogram based on different metrics. **(C)** The correlation among WGCNA gene modules and the nitrogen metabolites with highlighting target modules with red (positive correlation) or green (negative correlation).

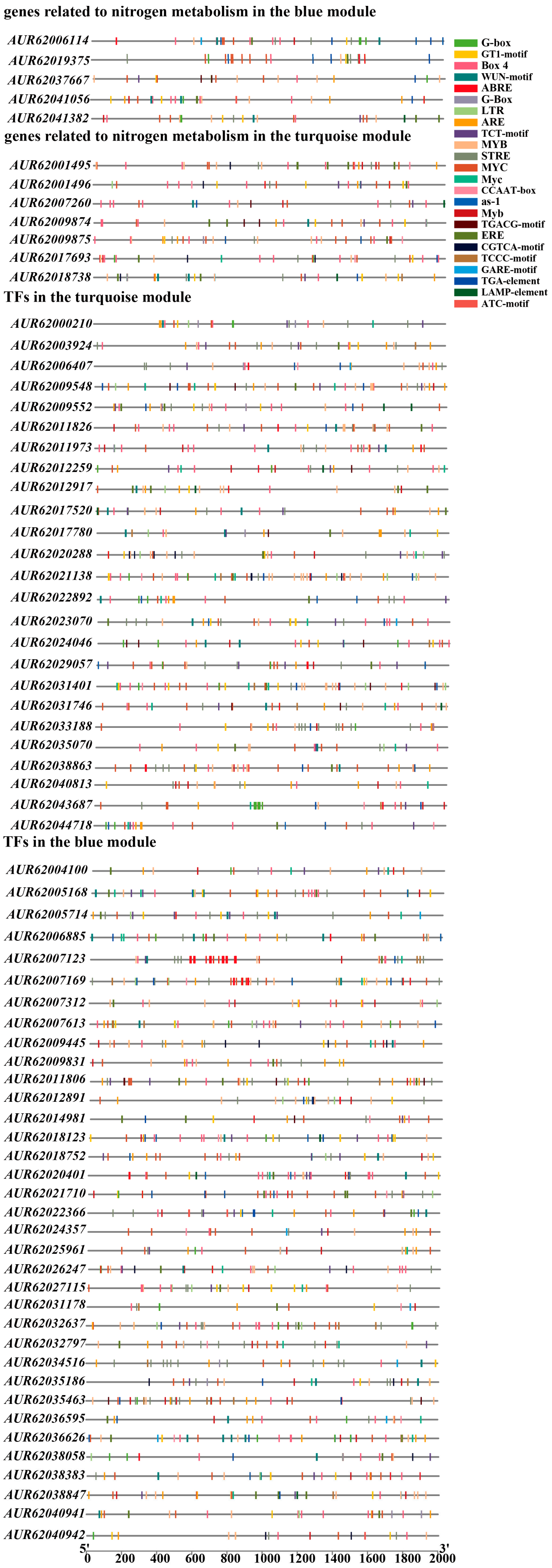
genes related to nitrogen metabolism in the blue module
AUR62006114
AUR62019375
AUR62037667
AUR62041056
AUR62041382
genes related to nitrogen metabolism in the turquoise module
AUR62001495
AUR62001496
AUR62007260
AUR62009874
AUR62009875
AUR62017693
AUR62018738
TFs in the turquoise module
AUR62000210
AUR62003924
AUR62006407
AUR62009548
AUR62009552
AUR62011826
AUR62011973
AUR62012259
AUR62012917
AUR62017520
AUR62017780
AUR62020288
AUR62021138
AUR62022892
AUR62023070
AUR62024046
AUR62029057
AUR62031401
AUR62031746
AUR62033188
AUR62035070
AUR62038863
AUR62040813
AUR62043687
AUR62044718
TFs in the blue module
AUR62004100
AUR62005168
AUR62005714
AUR62006885
AUR62007123
AUR62007169
AUR62007312
AUR62007613
AUR62009445
AUR62009831
AUR62011806
AUR62012891
AUR62014981
AUR62018123
AUR62018752
AUR62020401
AUR62021710
AUR62022366
AUR62024357
AUR62025961
AUR62026247
AUR62027115
AUR62031178
AUR62032637
AUR62032797
AUR62034516
AUR62035186
AUR62035463
AUR62036595
AUR62036626
AUR62038058
AUR62038383
AUR62038847
AUR62040941
AUR62040942
5'
3'
0
200
400
600
800
1000
1200
1400
1600
1800
2000
G-box
GT1-motif
Box 4
WUN-motif
ABRE
G-Box
LTR
ARE
TCT-motif
MYB
STRE
MYC
Myc
CCAAT-box
as-1
Myb
TGACG-motif
ERE
CGTCA-motif
TCCC-motif
GARE-motif
TGA-element
LAMP-element
ATC-motif

Figure S3. The cis-active elements prediction of all N-related genes and TFs promoters was performed using TBtools.

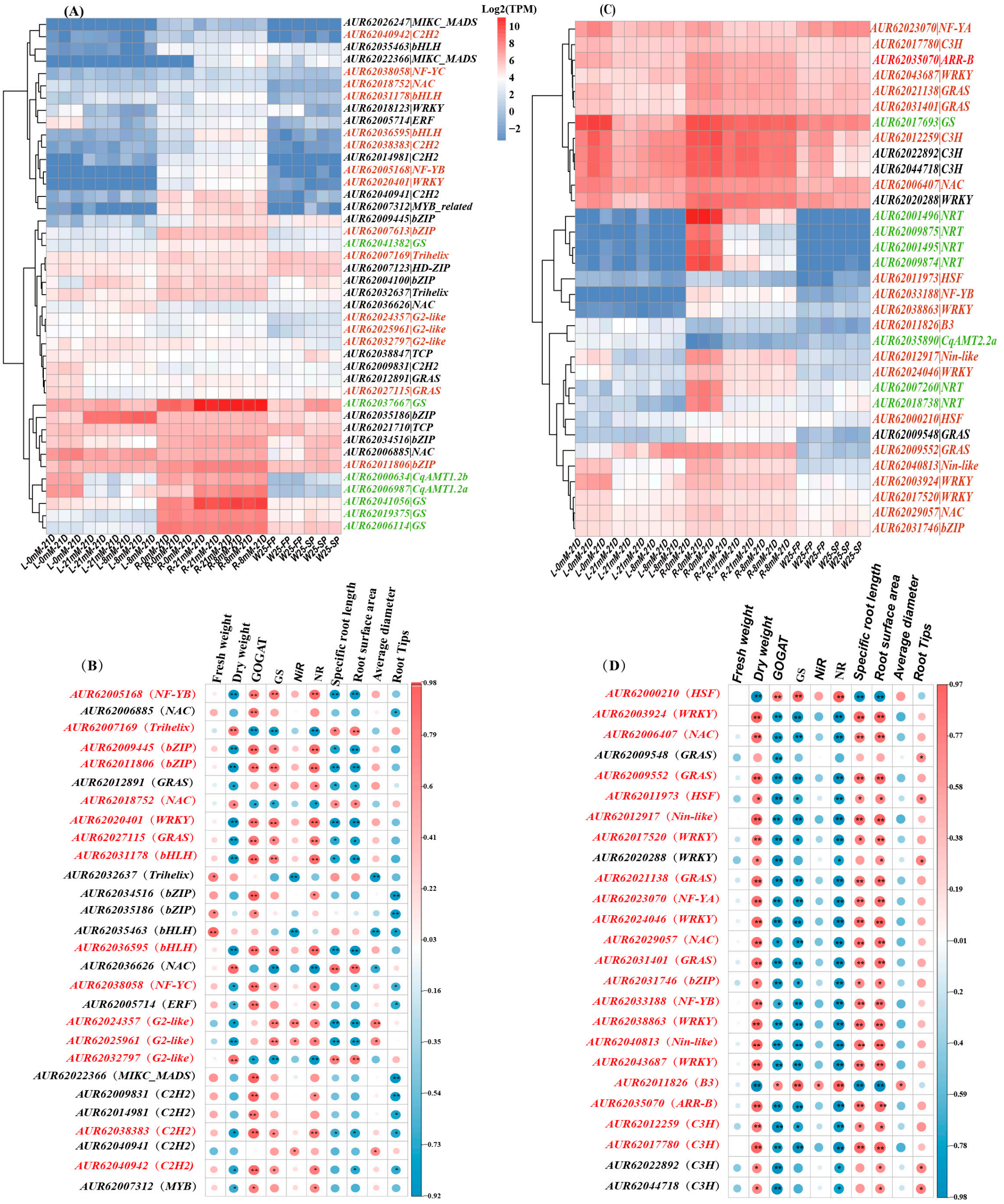

Figure S4. Identification of the TFs and genes related to nitrogen metabolism in the blue and turquoise module. **(A)** The blue module indicates the expression levels of selected TFs and nitrogen metabolism-related genes in different tissues and samples with different nitrogen concentrations. (L-0mM-21D—Leaf samples were treated with 0mM ammonium nitrogen for 21 days, with similar descriptions as those below for L-21mM-21D and L-8mM-21D; R-0mM-21D—Root samples were treated with 0mM ammonium nitrogen for 21 days,with similar descriptions as below for R-21mM-21D and R-8mM-21D;W25-FP—panicles of W25 at the flowering stage; W25-SP—panicles of W25 at the seed formation stage)**(B)** The turquoise module indicates the expression levels of selected TFs and nitrogen metabolism-related genes in different tissues and samples with different

nitrogen concentrations. **(C)** The pearson's correlation analysis between the key TFs expression and changes of physiological traits in the blue module. **(D)** The pearson's correlation analysis between the key TFs expression and changes of physiological traits in the turquoise module. The * symbol represents $0.01 < P < 0.05$. The ** symbol represents $P < 0.01$.

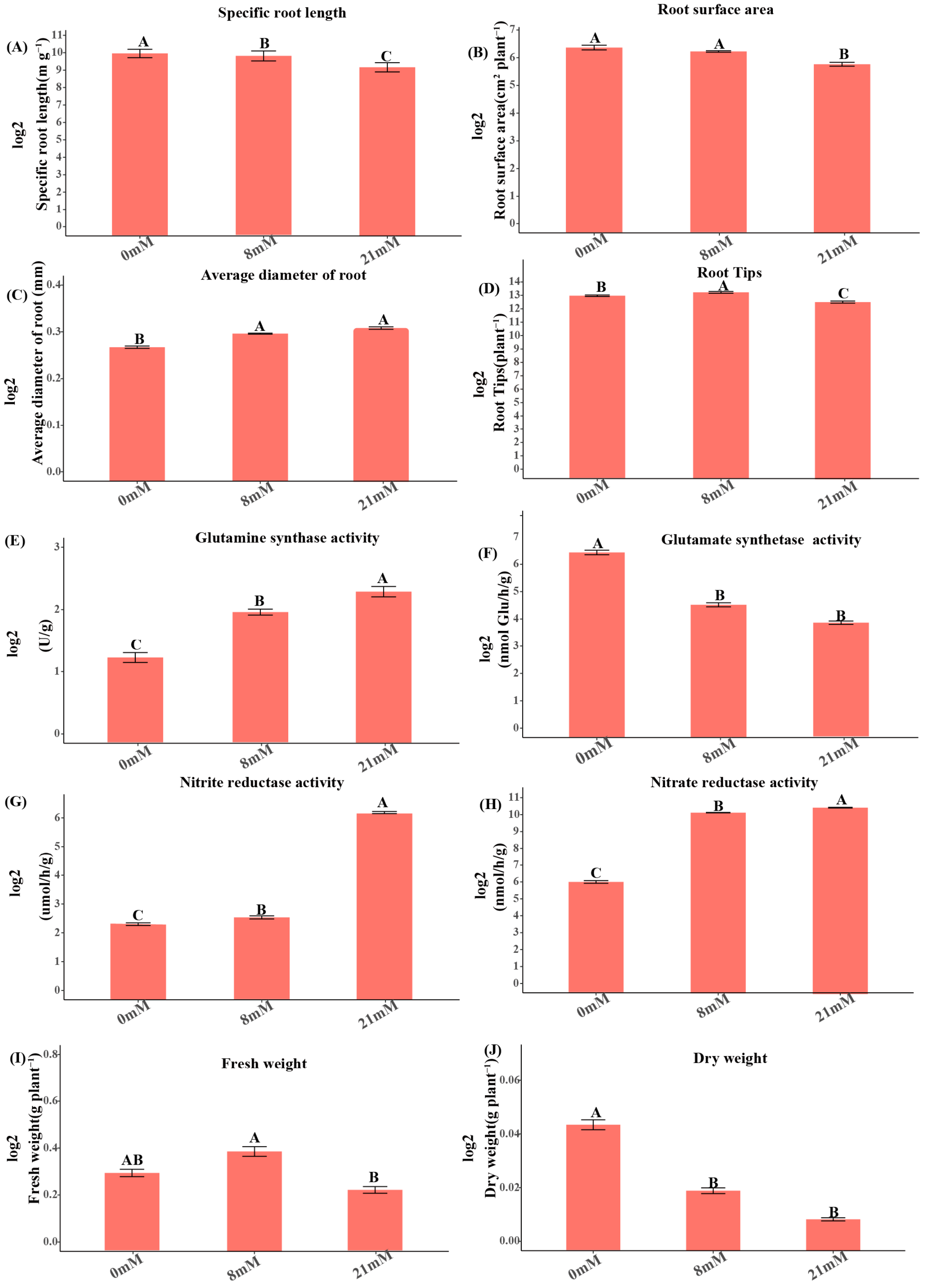

Figure S5. Measurement of physiological characteristics of quinoa root. Treatments with different capital letters are significantly different at $p < 0.05$. **(A)** Specific root length. **(B)** Root surface area. **(C)** Average diameter of root. **(D)** Number of root tips. **(E)** Glutamine synthase activity. **(F)**

Glutamate synthetase activity. **(G)** Nitrite reductase activity. **(H)** Nitrate reductase activity. **(I)** Fresh weight. and **(J)** Dry weight.

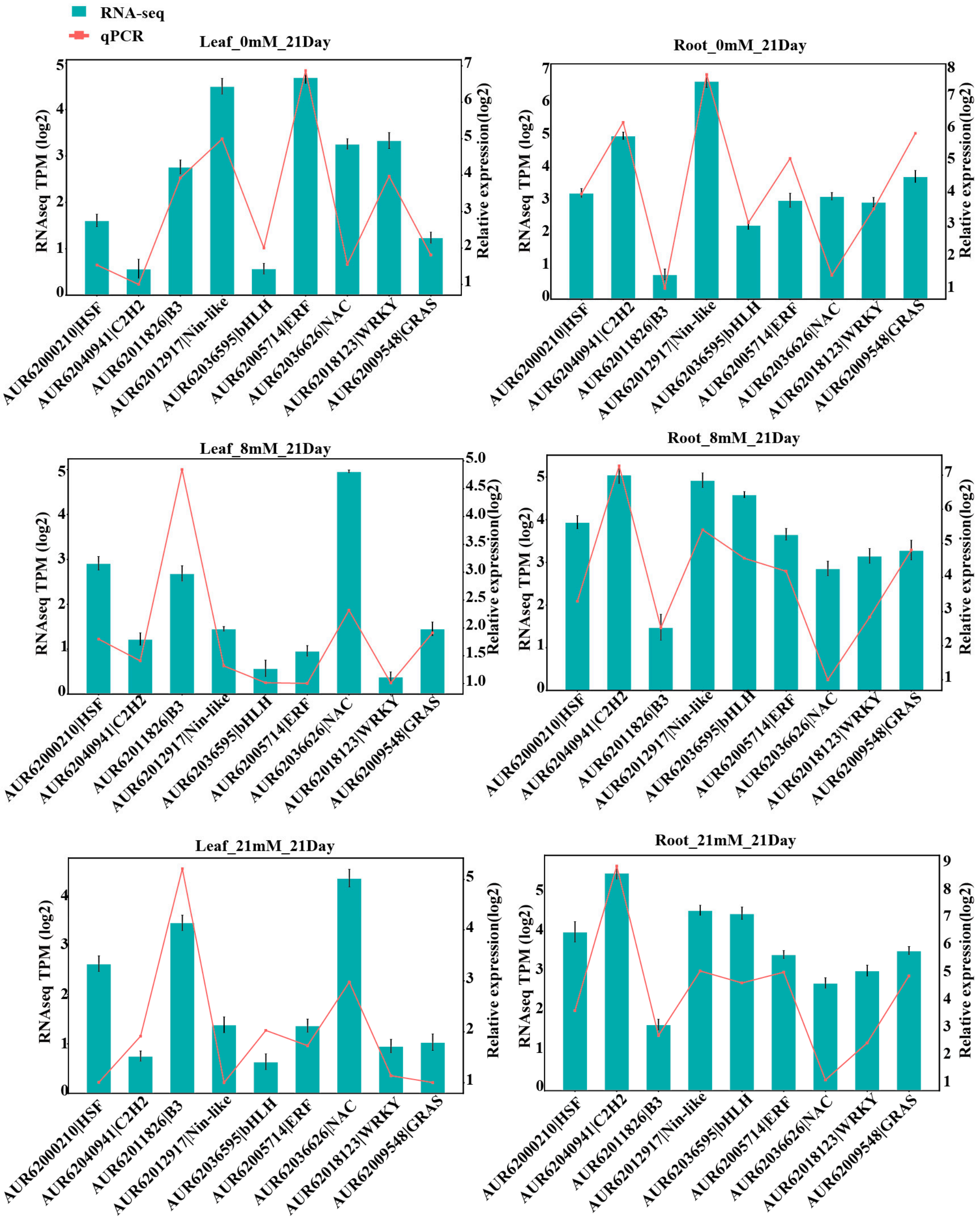

Figure S6. qRT-PCR analysis of the 9 *TFs* in both leaves and roots under hydroponic cultivation of W32 after 21 days with 0, 8, and 21 mMNH_4^+ concentrations.

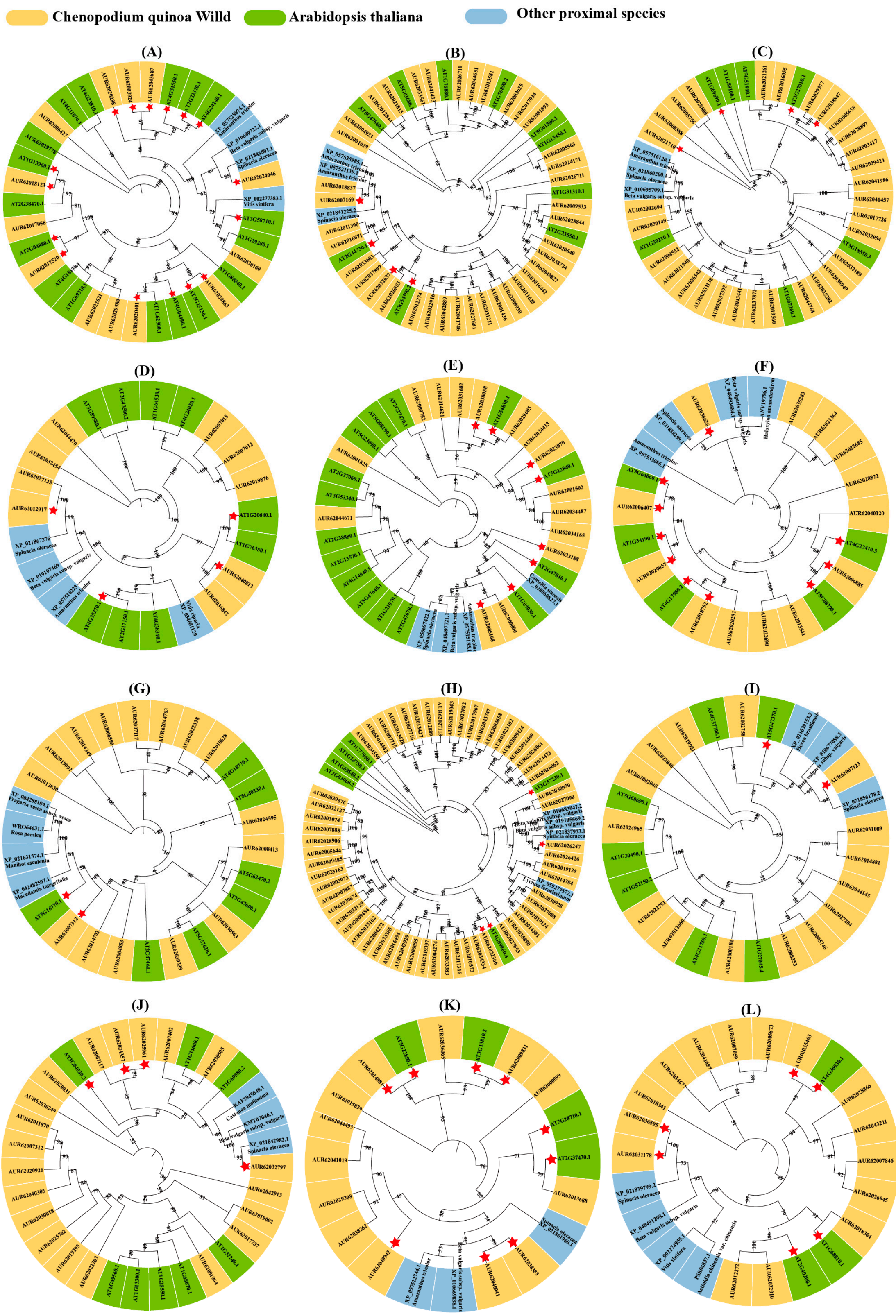

Figure S7. Phylogenetic tree of 12 transcription factors gene family of quinoa. **(A)** *WRKY* gene family. **(B)** *Trihelix* gene family. **(C)** *TCP* gene family. **(D)** *NLP* gene family. **(E)** *NF-Y* gene family. **(F)** *NAC* gene family. **(G)** *MYB* gene family. **(H)** *MADS-box* gene family. **(I)** *HD-ZIP* gene family. **(J)** *G2-like* gene family. **(K)** *C2H2* zinc finger gene family. **(L)** *bHLH* gene family.
